# The Association Between Tobacco Use and Risk of COVID-19 Infection and Clinical Outcomes in Sweden: A Population-Based Study

**DOI:** 10.3389/ijph.2023.1606175

**Published:** 2023-11-22

**Authors:** A. N. Shaaban, F. Andersson, S. Peña, I. H. Caspersen, C. Magnusson, N. Orsini, S. Karvonen, P. Magnus, M. P. Hergens, M. R. Galanti

**Affiliations:** ^1^ Department of Global Public Health, Karolinska Institutet, Stockholm, Sweden; ^2^ Centre for Epidemiology and Community Medicine, Stockholm, Sweden; ^3^ Finnish Institute for Health and Welfare, Helsinki, Finland; ^4^ Centre for Fertility and Health, Norwegian Institute of Public Health, Oslo, Norway; ^5^ Unit for Communicable Disease Control, Stockholm, Sweden; ^6^ Infectious Diseases Unit, Department of Medicine Solna, Karolinska Institutet, Solna, Sweden

**Keywords:** smoking, Sweden, COVID-19, smokeless tobacco (*snus*), population-based cohort

## Abstract

**Background:** The association between tobacco use and COVID-19 is controversial. During the early course of the pandemic, limited testing prevented studying a wide spectrum of clinical manifestations.

**Objective:** To examine the potential causal association between tobacco use and COVID-19 during the second wave (1 October 2020–30 June 2021) of the pandemic in Stockholm, Sweden.

**Methods:** A population-based cohort study was conducted in the Stockholm region of Sweden, with information on tobacco use collected prior to the pandemic. Adjusted relative risks (RR) of COVID-19 and 95% confidence intervals (CI) were calculated, contrasting current smokers and *snus* users to non-users of tobacco.

**Results:** Compared with non-users of tobacco, current smokers had a lower risk of COVID-19 (RR 0.78, 95% CI = 0.75–0.81) and of hospitalisation for the disease. Current s*nus* users had a higher risk of COVID-19. Heavy smokers and *snus* users had longer hospital stays than non-users of tobacco.

**Conclusion:** Tobacco use may have a different impact on the risk of being infected with SARS-CoV-2 and the risk of developing severe clinical manifestations. Further research is needed to determine the underlying mechanisms.

## Introduction

COVID-19, caused by SARS-CoV-2 [1], has resulted in approximately 664 million cases and 7 million deaths globally by 24 January 2023 [2]. While tobacco smoking has been considered a risk factor due to its numerous toxic constituents [3] and the increased susceptibility to infections [4–6], some studies found an inverse association between smoking and SARS‐CoV‐2 infection risk [7, 8]. To elucidate the inverse relationship between smoking and COVID-19 risk, several hypotheses have been posited: one suggests that smoking could activate the aryl-hydrocarbon receptor (AHR), possibly suppressing the angiotensin-converting enzyme 2 (ACE2) receptor which the virus uses for cellular entry [9]; another underscores the potential of nicotine to modulate inflammation [10, 11]; and yet another speculates on nitric oxide’s toxic influence on the virus [12]. All these hypotheses remain speculative.

In fact, the reported reduced risk of COVID-19 among smokers may arise from information bias; confounding; and selection bias [13]. Further, studying samples with hospitalised patients or individuals tested for COVID-19, where testing has not been random, are more likely to be affected by selection or collider bias [14]. Smokers may be under-represented among individuals who undertook the SARS-CoV-2 RT-PCR test; therefore, the decreased risk would rather be due to under-diagnosis, especially in studies where the analysis was limited to tested individuals [15]. This possibility may be particularly relevant because of restricted access to COVID-19 testing during the first wave of the pandemic when testing was limited to symptomatic cases with high exposure risk and healthcare workers [16, 17]. Insufficient data on who is infected by the SARS-CoV-2 prevents a complete understanding of risk factors for COVID-19.

A source of bias that has not received enough attention so far is the non-differential misclassification of exposure, which usually entails a bias towards the null [18]. This may occur if the information on tobacco use was collected long before the outcome, because individuals may have changed their behaviour. For instance, in a recent article based on the UK Biobank, the median time between assessment of smoking and the pandemic was more than 9 years [19]. An appropriate study design that addresses these biases is required to study the association between tobacco use and COVID-19 in a reliable way.

A recent population-based cohort in Sweden based on cases occurring during the first wave of the pandemic found smokers to be at reduced risk of being diagnosed with COVID-19 after adjustment for several potential confounders, while the reverse was true concerning the use of the moist oral tobacco *snus* [20]. The characteristics of the second wave of the pandemic were quite different from the first one in terms of the spreading of the infection (higher in the second wave) and severity of the disease (less severe in the second wave) [21, 22]. Also, the Swedish SARS-CoV-2 testing policy underwent significant changes between the first and second wave of the pandemic. During the first wave the Swedish Public Health Agency, Folkhälsomyndigheten (FHM), recommended a restrictive approach [23], allowing testing only on medical prescription, and limited to hospitalised individuals and those with COVID-19 symptoms or recent travel history to high-risk areas. However, as the pandemic advanced, evidence emerged that asymptomatic individuals could also transmit the virus [24]. Therefore, starting from June 2020, Sweden’s policy shifted towards a more liberal approach, allowing testing on demand, as well as for individuals with mild COVID-19 symptoms or individuals in close contact with a confirmed COVID-19 case [25].

The temporal concentration of studies examining the association between tobacco use and COVID-19 during the initial wave represents a limitation given the potential bias due to testing opportunities, as a consequence of the prevailing testing policies. Moreover, analyses of adverse disease outcomes during the first wave may have been hampered by insufficient data. Therefore, this study aims to examine the association between tobacco use and COVID-19 and its clinical outcomes during the second wave of the pandemic in Sweden (1 October 2020–30 June 2021).

## Methods

### Study Population and Analytical Sample

**FIGURE 1:**
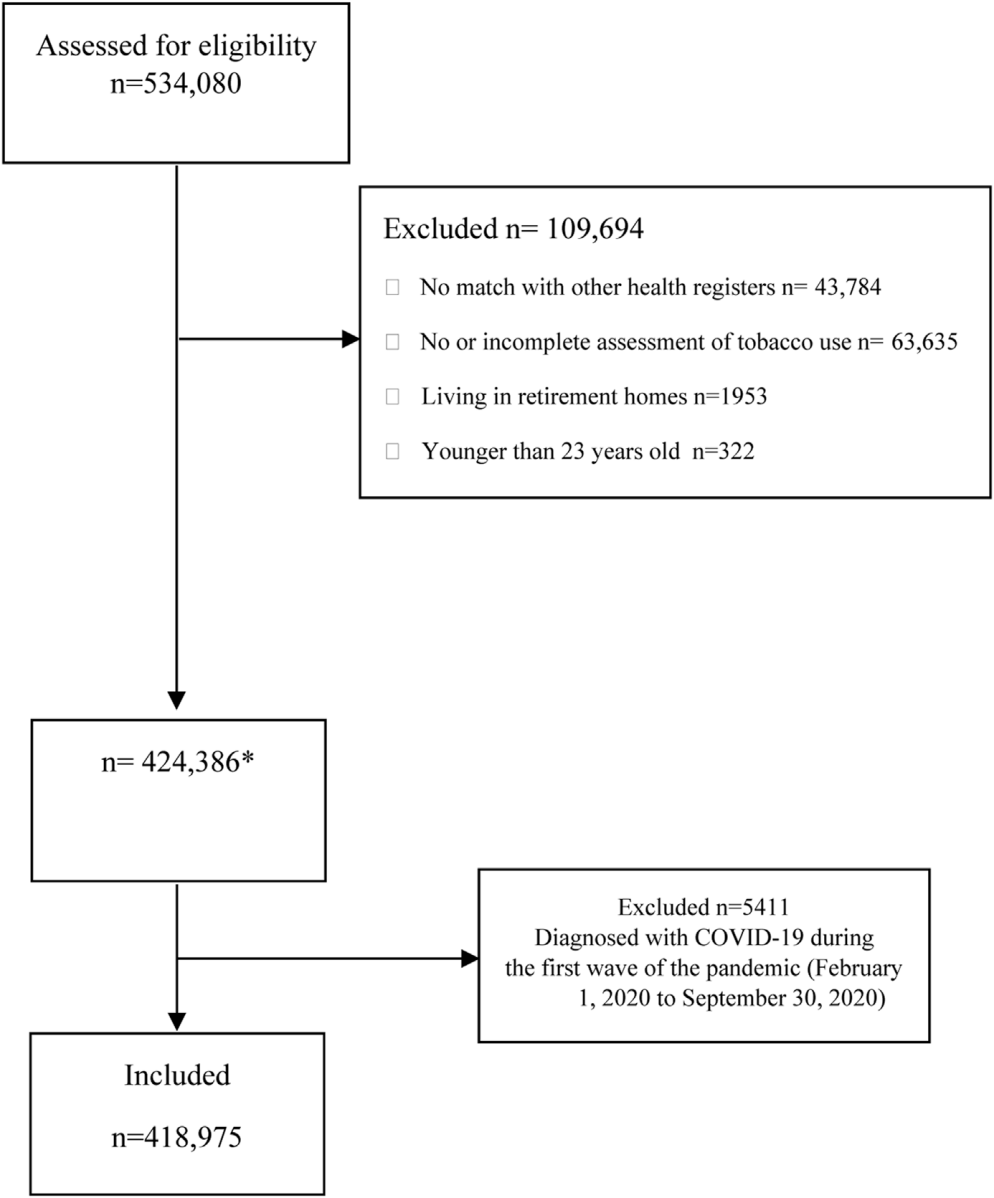
Flow diagram of participants (The Association Between Tobacco Use and Risk of COVID-19 Infection and Clinical Outcomes in Sweden: A Population-Based Study, Stockholm, Sweden, 2023).

We used medical records of adult clients aged 23 or older who sought care at public dental clinics in the Stockholm region from October 2015 to January 2020. In Sweden, region-based public dental clinics, known as Folktandvården (FTV), provide preventive oral check-ups to all residents who choose to receive care at these facilities. Detailed information regarding the public dental care system in Stockholm region has been given in a prior publication [26]. During visits to the public dental clinics, information regarding smoking and *snus* use is regularly collected and classified into no or current use, the latter including also information on the amount habitually used. Using the national personal numbers assigned to each resident in Sweden, we linked this information to the regional inpatient and outpatient healthcare (VAL) database to obtain information on COVID-19 diagnoses among the study population. Demographic information was obtained through record-linkage with the register of the total population of the Stockholm region held by Statistics Sweden. Only individuals with complete information on tobacco use who could be linked to the regional records were considered for the analysis. Subjects with a confirmed diagnosis of COVID-19 up to 30 September 2020, and those residing in assisted elderly living facilities were excluded. The latter include individuals with high levels of disabilities, elevated mortality rates due to COVID-19 and other factors, and a likely low prevalence of tobacco use. After these exclusions, there were 418,975 individuals in the cohort. Figure 1 displays the derivation of the analytical sample.

### Variables

#### Exposure Variables (Current Tobacco Use)

Current tobacco use was assessed separately for cigarette smoking and *snus* use*.* Both current cigarette smoking and *snus* use were reported as dichotomous variables (yes/no) with additional categorical variables giving the average daily consumption (for current cigarette smoking: 10 cigarettes per day or less (CPD); more than 10 CPD; for *snus* use: less than 1/2 can per day; ½ can per day or more). Only individuals who reported the amount of tobacco use were included in the analyses. This meant that 2,108 individuals who reported current use but not its amount were excluded. Different categorisations of tobacco use into mutually exclusive categories, including dual users, were also used in the analysis.

Information about past tobacco use was found to be incomplete due to inconsistent assessment. Therefore, former users were included in the category of current non-users.

#### Outcome Variables (COVID-19 Diagnoses and Clinical Outcomes)

Information on COVID-19 diagnoses was accrued from healthcare registers from 1 October 2020 to 30 June 2021. Five outcome variables were considered: 1. any diagnosis of COVID-19, confirmed by a positive polymerase chain reaction (PCR) test reported to the regional surveillance unit; 2. hospital admission with a diagnosis of COVID-19, as recorded in the International Classification of Diseases 10th Revision (ICD-10) codes U071 and U072, either as the primary or a secondary cause of admission; 3. admission to an intensive care unit (ICU) due to a COVID-19 diagnosis, recorded with the ICD-10 codes mentioned above; 4. death by COVID-19, registered in the Swedish Cause of Death Registry based on the death certificate filled out by physicians. All deaths during the follow-up period with COVID-19 as the primary cause were included in the analysis; 5. the number of days of hospital stay among individuals admitted to a hospital with a diagnosis of COVID-19.

#### Other Covariates

Other variables included in the analysis as potential confounders were: sex; age (as continuous variable); country of birth (Sweden, other Nordic countries, or other countries); achieved education (compulsory school, high school, or university); occupational risk categorised as high, moderate, or low risk of SARS-CoV-2 infection based on the probability of disease transmission connected to a specific job title. For instance, healthcare workers were considered at high risk; teachers were considered at moderate risk; while clerks enabled to remote work were considered at low risk; household disposable income expressed in Swedish crowns per year; and cohabitation (labeled as “yes” for those living with others and “no” for individuals living alone).

### Statistical Analysis

Relative risks (RR) and their corresponding 95% confidence intervals (CI) of the study outcomes according to tobacco use were estimated using generalised linear models (GLMs). We used a modified Poisson regression with robust standard errors [27]. The study outcomes were considered both in a cumulative fashion (i.e., “any diagnosis” includes even hospitalised, intensive care and deaths) and as mutually exclusive events, i.e., extra-hospital diagnoses with non-lethal outcomes; hospital diagnoses with no intensive care; intensive care surviving the disease; deaths. In a primary analysis, we compared exclusive current users of either type of tobacco (i.e., cigarettes or *snus*) as mutually exclusive categories with current non-users of any tobacco. It should be noted that among these latter, some individuals may have been former tobacco users. Adjustments were made for putative confounders, namely, age, sex, country of birth, occupational risk, education, income, and cohabitation, selected using a direct acyclic graph as shown in a previous study [20]. Adjusted RR and 95% confidence intervals (CI) of diagnosis of COVID-19 are presented separately by sex in the analyses of *snus* use.

We conducted sensitivity analyses as follows: a) by using information on smoking and *snus* use ascertained in different periods before the pandemic (2015–17, 2018, 2019–20) in order to understand whether information bias due to non-differential exposure misclassification could have affected the results; b) by mutually adjusting the estimates for either tobacco product instead of excluding dual users; c) through analyses of mutually exclusive outcome events to assess whether the prognosis of COVID-19 was affected by tobacco use; d) through stratified analyses by time period with reference the roll out of the vaccination program (pre-vaccination: 1 October to 31 December 2020, and post-vaccination: 1 January to 30 June 2021).

A negative binomial regression count model [28] was used to assess the association between tobacco use and inpatient length of stay (number of days).

All analyses were conducted using Stata version 17 (StataCorp LP, College Station, Texas, United States) and R version 4.1.2.

## Results

**FIGURE 2:**
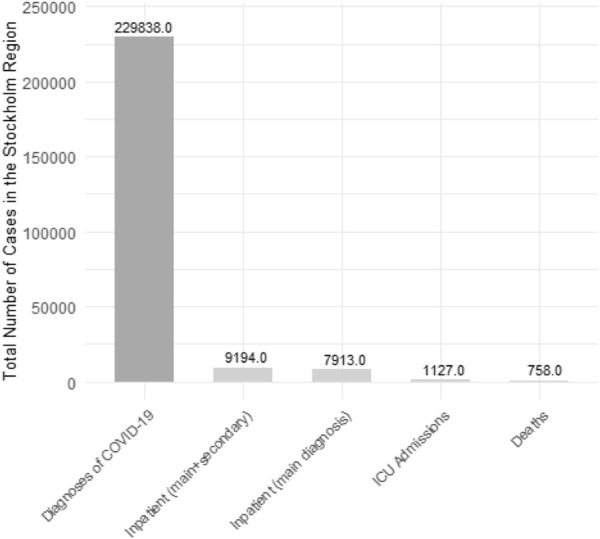
Total number of COVID-19-related cases, inpatient and ICU admissions, as well as deaths, in the Stockholm region. Source of data: The Public Health Agency of Sweden. For individuals aged 23 and above between October 2020 and June 2021, excluding those residing in retirement homes (The Association Between Tobacco Use and Risk of COVID-19 Infection and Clinical Outcomes in Sweden: A Population-Based Study, Stockholm, Sweden, 2023).

A total of 418,975 participants were included in the study. Table 1 shows the cohort participants’ baseline socio-demographic characteristics and tobacco use, and incident diagnoses of COVID-19, also in separate sub-cohorts identified by period of assessment of tobacco use. The proportion of current smokers was 9.6%, current *snus* users was 12.5%, and that of dual users was 1.2%. The cumulative incidence of COVID-19 diagnoses in the study period was 12.0% of hospital admissions 0.6%, of ICU 0.06%, and of death 0.05%. Table 1 illustrates the socio-demographic characteristics of the study population and the distribution of covariates across categories of current tobacco use. Figure 2 illustrates the cumulative incidence of COVID-19-related diagnoses, inpatient and ICU admissions, as well as deaths, in the Stockholm region for individuals aged 23 and above between October 2020 and June 2021, excluding those residing in retirement homes. Table 2 reports the distribution of incident COVID-19 diagnoses and disease outcomes (hospital admissions, admission to ICU, and deaths) across categories of current tobacco use.

**TABLE 1:** Socio-demographic characteristics, tobacco use and incident diagnoses of COVID-19 among clients of the public dental care clinics in Stockholm region during the second epidemic wave (between 1 October 2020–30 June 2021) (The Association Between Tobacco Use and Risk of COVID-19 Infection and Clinical Outcomes in Sweden: A Population-Based Study, Stockholm, Sweden, 2023).

	Total *N* = 418,975	Non-tobacco users (all)	Smokers (all)	Non-tobacco users (males)	*Snus* users (males)	Non-tobacco users (females)	*Snus* users (females)
	N (%)	N (%)	N (%)	N (%)	N (%)	N (%)	N (%)
Sex							
Male	228,953 (54.7)	137,820 (41.1)	16,245 (45.6)				
Female	190,022 (45.3)	197,544 (58.9)	19,404 (54.4)	—	—	—	—
Age mean (SD)	47.23 (15.8)	47.9 (16.1)	45.5 (15.5)	48.3 (16.0)	43.7 (13.4)	47.6 (16.1)	42.2 (12.8)
Education							
Compulsory (9 years)	34,087 (8.2)	23,779 (7.2)	6,206 (17.7)	10,545 (7.75)	3,838 (10.6)	13,234 (6.8)	595 (5.51)
High school (12 years)	141,015 (34.1)	103,720 (31.4)	17,558 (50.0)	45,646 (33.6)	16,735 (46.0)	58,074 (29.8)	3,782 (35.03)
University (>12 years)	234,507 (56.7)	200,225 (60.5)	10,941 (31.2)	78,386 (57.6)	15,637 (43.0)	121,839 (62.5)	6,396 (59.23)
Unknown	3,675 (0.9)	3,100 (0.9)	380 (1.1)	1,419 (1.0)	151 (0.4)	1,681 (0.9)	25 (0.23)
Occupational risk for infection							
Low	253,567 (60.5)	209,276 (62.4)	17,089 (47.9)	91,177 (66.2)	20,642 (56.1)	118,099 (59.8)	5,596 (51.0)
Moderate	75,227 (18.0)	55,552 (16.6)	8,791 (24.7)	22,781 (16.5)	9327 (25.4)	32,771 (16.6)	2045 (18.7)
High	54,708 (13.1)	42,846 (12.8)	6,254 (17.5)	10,769 (7.8)	3,289 (8.9)	32,077 (16.2)	2,472 (22.6)
Unknown	35,473 (8.4)	27,690 (8.3)	3,515 (9.9)	13,093 (9.5)	3,524 (9.6)	14,597 (7.4)	849 (7.7)
Disposable yearly income in Swedish crowns, mean (SD)	270,062.6 (409,889.8)	273,763.1 (39,114.6)	224,631.2 (342,944.3)	289,933.1 (46,080.5)	280,357.6 (586,055.3)	262,499.6 (333,689.5)	25,803.4 (420,319.4)
Cohabitation							
Yes	325,487 (78.7)	263,771 (79.6)	24,673 (70.7)	107,524 (79.1)	28,150 (77.2)	156,247 (80.0)	8,221 (75.5)
No	88,251 (21.3)	67,509 (20.4)	10,205 (29.3)	28,358 (20.9)	8,297 (22.8)	39,151 (20.0)	2,672 (24.5)
Country of birth							
Sweden	332,421 (80.4)	264,390 (79.9)	25,373 (72.6)	110,799 (81.6)	32,951 (90.3)	153,591 (78.7)	10,103 (92.7)
Other Nordic Countries	11,813 (2.9)	9,770 (2.9)	1,114 (3.2)	2,974 (2.2)	624 (1.7)	6,796 (3.5)	250 (2.3)
Other countries	69,261 (16.7)	56,781 (17.2)	8,475 (24.2)	22,046 (16.2)	2,908 (8.0)	34,735 (17.8)	545 (5.0)
Diagnoses of COVID-19							
No	368,471 (87.9)	294,977 (88.0)	32,200 (90.33)	121,705 (88.31)	31,767 (86.37)	173,272 (87.71)	9,440 (86.12)
Yes	50,504 (12.1)	40,387 (12.0)	3,449 (9.67)	16,115 (11.69)	5,015 (13.63)	24,272 (12.29)	1,522 (13.88)
Hospital admissions with COVID-19							
No	416,608 (99.4)	333,374 (99.4)	35,503 (99.6)	136,776 (99.2)	36,591 (99.5)	196,598 (99.5)	10,929 (99.7)
Yes	2,367 (0.6)	1,990 (0.6)	146 (0.4)	1,044 (0.8)	191 (0.5)	946 (0.45)	33 (0.3)
Hospital intensive care for COVID-19							
No	418,729 (99.9)	335,162 (99.9)	35,632 (99.95)	137,697 (99.91)	36,757 (99.9)	197,465 (99.96)	10,959 (99.97)
Yes	246 (0.1)	202 (0.1)	17 (0.05)	123 (0.09)	25 (0.1)	79 (0.04)	3 (0.03)
Death with a diagnosis of COVID-19							
No	418,754 (99.9)	335,163 (99.94)	35,639 (99.97)	137,698 (99.91	174,471 (99.92	197,465 (99.96)	10,962 (100.00)
Yes	221 (0.1)	201 (0.06)	10 (0.03)	122 (0.09)	131 (0.08)	79 (0.04)	0 (0.0)

**TABLE 2:** Adjusted Relative Risk (RR) and 95% Confidence Intervals (CI) of increasing length of hospital stay (in days) for current exclusive smokers and exclusive *snus* users compared to non-tobacco users admitted to hospital because of COVID-19, among clients of the public dental care clinics in Stockholm region during the second epidemic wave (1 October 2020–30 June 2021) (The Association Between Tobacco Use and Risk of COVID-19 Infection and Clinical Outcomes in Sweden: A Population-Based Study, Stockholm, Sweden, 2023).

	Number of patients who needed in-patient care by tobacco use status	Length of stay (days)
	*N* (%)	Adjusted^a^ RR (95% CI)
Current tobacco Use		
Non-User	1,990 (93.2)	(ref)
Smoker (all)	146 (6.9)	1.18 (0.99–1.41)
≤10 cig/day	103 (4.8)	1.10 (0.89–1.35)
>10 cig/day	43 (2.0)	1.37 (1.01–1.84)
Current tobacco Use		
Non-User	1,990 (89.9)	(ref)
*Snus* user (all)	224 (10.1)	1.08 (0.93–1.25)
<1/2 can/day	132 (6.0)	0.93 (0.77–1.12)
>1/2 cans/day	92 (4.2)	1.29 (1.04–1.60)

^a^ Adjusted for sex, age, education, income, occupational risk, country of birth, and cohabitation.

**FIGURE 3:**
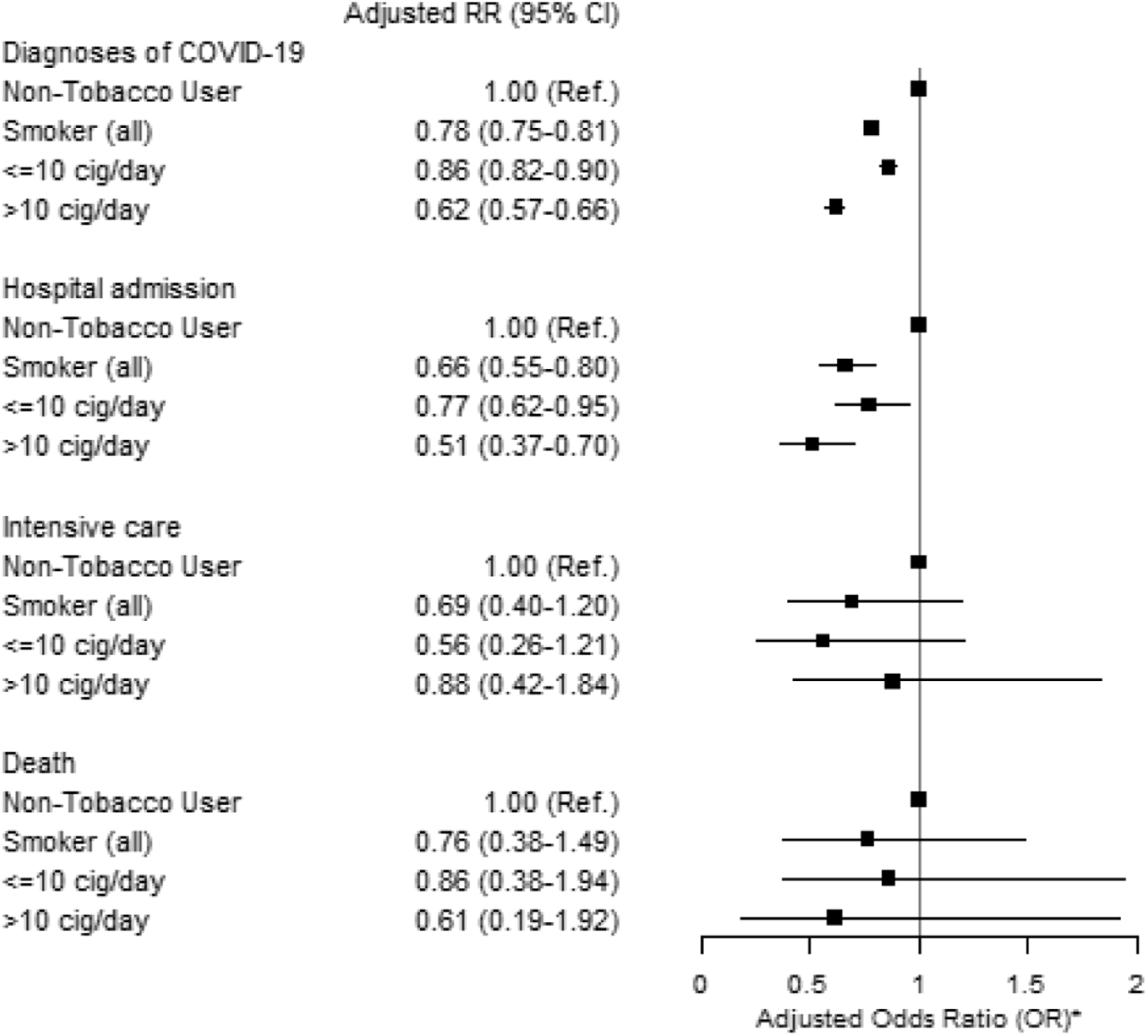
Adjusted Relative Risk (RR) and 95% Confidence Intervals (CI) of diagnosis and severity of COVID-19 for current exclusive smokers compared with non-tobacco users among clients of the public dental care clinics in Stockholm region during the second epidemic wave (1 October 2020–30 June 2021).* Adjusted for sex, age, education, income, occupational risk, country of birth and cohabitation (The Association Between Tobacco Use and Risk of COVID-19 Infection and Clinical Outcomes in Sweden: A Population-Based Study, Stockholm, Sweden, 2023).

Current exclusive smokers had a lower risk of being diagnosed with COVID-19 than current non-users of tobacco (adjusted RR 0.78, 95% CI = 0.75–0.81) (Figure 3). Current exclusive smokers also had a lower risk of being admitted to a hospital due to COVID-19 than current non-tobacco users (adjusted RR 0.66, 95% CI = 0.55–0.80) (Figure 3). The results were unchanged when including dual tobacco users and adjusting for *snus* use (Supplementary Table S1); and were very similar in separate analyses by sex (not shown).

**FIGURE 4:**
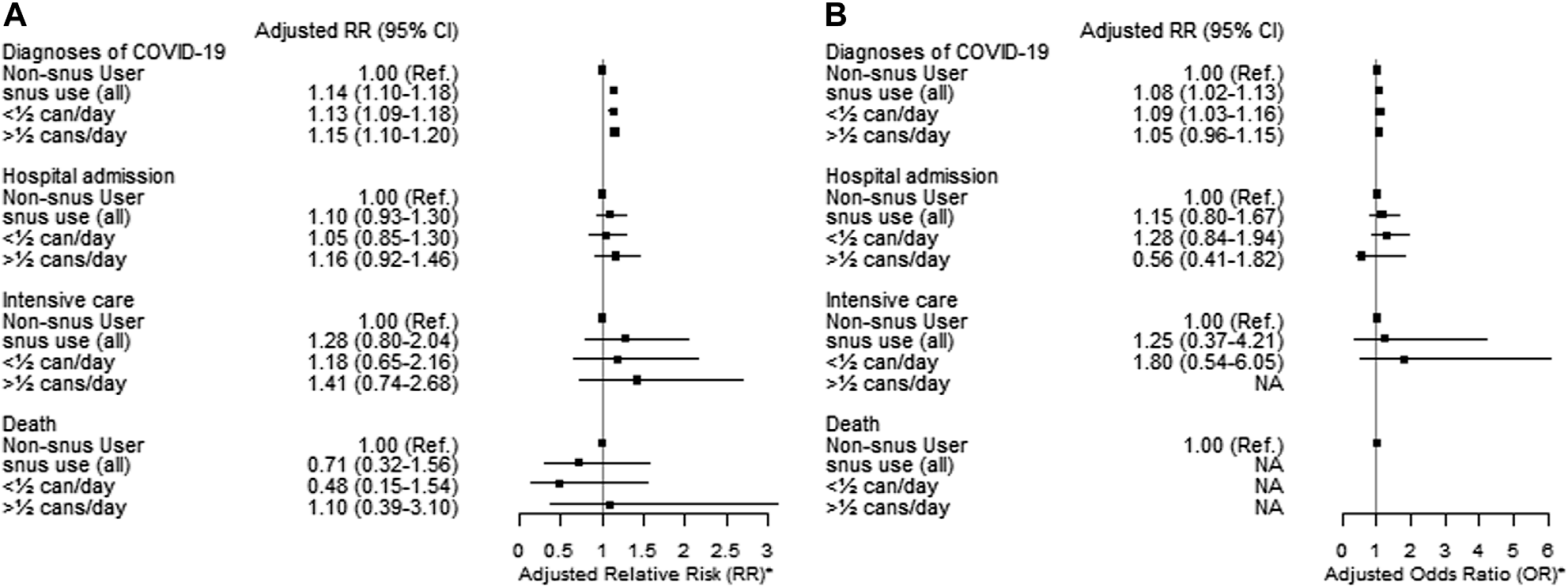
Adjusted Relative Risk (RR) and 95% Confidence Intervals (CI) of diagnoses of COVID-19 stratified by sex [males **(A)** and females **(B)**] for current exclusive *snus* users compared to non-users of tobacco among clients of the public dental care clinics in Stockholm region during the second pandemic wave (1 October 2020–30 June 2021). * Adjusted for age, education, income, occupational risk, country of birth and cohabitation (The Association Between Tobacco Use and Risk of COVID-19 Infection and Clinical Outcomes in Sweden: A Population-Based Study, Stockholm, Sweden, 2023).

Current exclusive *snus* users had a higher risk of being diagnosed with COVID-19, both among females (adjusted RR 1.08, 95% CI = 1.02–1.13) and males (adjusted RR 1.14, 95% CI = 1.10–1.18) compared to non-users of tobacco (Figure 4). The results were unchanged when including dual tobacco users and cigarette smoking (Supplementary Table S2).

A different categorisation of tobacco uses into mutually exclusive categories, including dual users, yielded very similar results (Supplementary Table S3). Sensitivity analyses of COVID-19 risk according to tobacco use assessed in different periods did not yield results different from those obtained with the whole cohort. If anything, a slightly stronger inverse association with smoking was seen in the less recently assessed cohort (Supplementary Tables S4, S5).

A positive association emerged between exclusive smoking and length of stay in hospital compared to non-tobacco use, particularly among smokers of >10 cig/day (adjusted RR = 1.37, 95% CI = 1.01–1.84) (Table 2). The same pattern was observed among exclusive *snus* users (adjusted RR among users of ≥½ can/day = 1.29, 95% CI = 1.04–1.60) (Table 2).

Using mutually exclusive outcome events, we found that the risk of being diagnosed with COVID-19 that did not result in hospital admission, admission to ICU, or death was lower among current exclusive smokers (adjusted RR = 0.79, 95% CI 0.76–0.82) and dual tobacco users (adjusted RR = 0.81, 95% CI = 0.75–0.89) compared to non-tobacco users (Supplementary Table S6). The opposite was observed among exclusive current *snus* users (adjusted RR = 1.13, 95% CI = 1.10–1.16) (Supplementary Table S6). The estimates of relative risks of hospital admission, ICU admission, and death were very imprecise and in general compatible with no association. A sensitivity analysis comparing pre- and post-vaccination periods showed associations consistent with the main findings (Supplementary Table S7). The associations with *snus* use were generally similar before and after the rollout of COVID-19 vaccinations (Supplementary Table S8). However, an indication of increased risk of hospital admission among men heavy *snus* users (adjusted RR = 1.42, 95% CI = 1.01–2.01) was revealed in the pre-vaccination period but not after the rollout of the vaccination program (adjusted RR = 0.98, 95% CI = 0.72.1.33).

## Discussion

This large population-based cohort examined the association between tobacco use and COVID-19 risk and its clinical outcomes during the second wave of the pandemic in Sweden (1 October 2020–30 June 2021). The cumulative incidence of COVID-19 diagnoses in this study (12.05%) was higher than the incidence reported during the first wave of the pandemic (1.5%), but mortality was similar in the two periods (0.05%) [20]. The 50,504 diagnosed COVID-19 cases in our study constitute 21% of the total reported COVID-19 cases (*N* = 229,838) in the Stockholm region during the corresponding study period. The cumulative incidence of COVID-19-related diagnoses, inpatient care, ICU admissions, and deaths in our cohort, 12.0%, 0.6%, 0.06%, 0.05%, respectively, closely mirrors that of the entire Stockholm region during the same study period, at 13.5%, 0.5%, 0.06%, and 0.04%, respectively. Current exclusive smoking was associated with a lower risk of COVID-19 infection and hospital admission after controlling for potential confounders, while *snus* use was associated with an increased risk of COVID-19 infection, in line with what was previously reported for the first wave of the pandemic in the same cohort [20]. In sensitivity analyses conducted to test the robustness of the main findings, these associations remained consistent and were not impacted by potential misclassification introduced by distant assessment of the exposure or by risk modification after the rollout of the vaccination program. Interestingly, the likelihood of progression of the disease using mutually exclusive outcome events, we found that the negative association with smoking was confined to diagnoses that did not require hospital admission and were not exiting in a death, indicating that smoking and *snus* use is potentially causally associated to the risk of infection rather than to the risk of disease progression. Also, when admitted to a hospital, current smokers, particularly heavy smokers, had prolonged hospital stays compared to non-users of tobacco. This finding indicates that smoking, a known cause of impairment of the respiratory system, could lead to more severe clinical course of COVID-19 in patients requiring hospital admission.

The association between cigarette smoking and the risk of COVID-19 has been assessed in several studies with mixed findings. Most studies that reported lower proportions of smokers among COVID-19 patients suffered from selection bias or bias introduced by limited testing opportunities during the first wave of the pandemic [14]. Although we cannot exclude an under-estimation of infections with SARS-CoV-2 among smokers, due to differences in testing behavior, the consistent findings during the two waves of the pandemic [20, 29], despite different testing scales and strategies, makes this potential bias unlikely. The inverse association between cigarette smoking and COVID-19 in our study, known also as “the smokers paradox,” matches the results from a review and meta-analysis in which current smokers had a lower risk of COVID-19 than non-smokers, similar to that found in this study [7]. However, it is also important to mention that other population-based studies found a positive association between smoking and COVID-19 diagnosis and disease progression. For example, a recent study based on data from the UK Biobank used a Mendelian randomisation (MR) analysis and found smoking to be associated with an increased risk of COVID-19 infection and of adverse disease outcomes [19]. However, the results from this study should be interpreted with caution as infection with SARS-CoV-2 and COVID-19 disease could also be predicted through risk-taking and impulsive behaviours, for instance, disregarding public health authorities’ prescriptions about distancing, face masks, etc. In fact, a previous study based on information from the UK biobank found that the candidate genes predicting smoking also predicted risk-taking behaviours [30].

*Snus* use was associated with a higher risk of COVID-19 infection in this study, as it was in an earlier study based on the same population [20]. This smokeless tobacco product is characterised by high level of nicotine absorption. Thus, the findings from these studies do not agree with the proposed beneficial role of nicotine against the SARS-COV2 virus. The fact that *snus* is primarily used in Sweden, and to a lesser extent in Finland and Norway, but banned by law across the rest of the European Union [31] gives these countries a unique position in contributing to the scientific knowledge on the possible role of nicotine. In fact, the results on *snus* in this study aligns with a parallel population-based study in Finland [32]. In addition, it should be noted that the elevated risk of hospitalisation observed among heavy *snus* users in our study was no longer present following the implementation of the COVID-19 vaccination campaign in Sweden. This finding underscores the crucial role of COVID-19 vaccination in mitigating the risk of severe outcomes among tobacco users.

The main limitation of this study is the lack of information on the incidence of testing among tobacco users. In fact, a lower incidence of PCR tests among smokers tests could at least partly explain the negative association found in this and other studies. However, consistent findings during the two waves of the pandemic in Sweden, despite different testing strategies, reduce the likelihood of a potential bias due to testing opportunities. The inclusion of former tobacco users among the current non-users may have biased downward the association introducing the potential for misclassification bias. Also, data in our study comes from individuals who underwent health checks at public dental clinics, who may not provide a representative sample of the source population. Notwithstanding, this study has several strengths. First, unlike other studies enrolling clinical samples, this study is based on a population-based cohort with the assessment of tobacco use completely prospective regarding the individual outcomes. Therefore, this study is less likely to be affected by selection or collider bias. Second, confounding was minimised by adjusting for several potential confounders that were unavailable in previous studies, such as occupational risk. Third, the study included several sensitivity analyses to test the stability of the results. Finally, to the best of our knowledge, this study included the largest analysis of COVID-19 among *snus* users, thus enabling further insights into the potential role of nicotine in the occurrence and prognosis of the COVID-19 disease.

### Conclusion

Smokers may be at lower risk of infection with SARS-CoV-2 but at higher risk of severe clinical course of COVID-19. The higher risk for both infection and severe clinical course among *snus* users does not support the hypothesised protective effect of nicotine on the disease. While the smoker’s paradox sparks interest, it is crucial to contextualize such findings within the broader scope of individual health. Smoking has long been established as a leading cause of numerous serious health conditions. From a public health perspective, the overwhelming adverse health consequences of tobacco use can far outweigh any potential reduction of SARS-CoV-2 infection risk.
