# Supplementary material for: The Association Between Tobacco Use and Risk of COVID-19 Infection and Clinical Outcomes in Sweden: A Population-Based Study
Source: Int J Public Health. 2023 Nov 22;68:1606175. doi: 10.3389/ijph.2023.1606175 (PMC10720900; doi:10.3389/ijph.2023.1606175)
Supplement: Supplementary file 1 [file DataSheet1.PDF]

SUPPLEMENTARY MATERIAL

The association between tobacco use and risk of COVID-19 infection and clinical outcomes during the second wave of the pandemic in Sweden: A population-based study

Supplementary Table S1. Adjusted Relative Risk (RR) and 95% Confidence Intervals (CI) of COVID-19 for current smokers compared to non-smokers among clients of the public dental care clinics in Stockholm region during the second epidemic wave (October 1, 2020 – June 30, 2021).

		All diagnoses of COVID-19	Hospital admission	Intensive care	Death
		Adjusted[a] RR (95% CI)	Adjusted[a] RR (95% CI)	Adjusted[a] RR (95% CI)	Adjusted[a] RR (95% CI)
**Current tobacco Use**					
Non-smoker		(ref)	(ref)	(ref)	(ref)
Smoker (all)		0.78 (0.75–0.81)	0.67 (0.55-0.80)	0.70 (0.41-1.22)	0.76 (0.39-1.49)
<=10 cig/day		0.86 (0.83–0.90)	0.79 (0.64-0.98)	0.62 (0.31- 1.23)	0.89 (0.41-1.93)
>10 cig/day		0.61 (0.57-0.65)	0.48 (0.35 - 0.67)	0.83 (0.39-1.77)	0.58 (0.18-1.86)

[a] Adjusted for *snus* use, sex, age, education, income, occupational risk, country of birth and cohabitation

Supplementary Table S2. Adjusted Relative Risk (RR) and 95% Confidence Intervals (CI) of COVID-19 stratified by sex for current *snus* users compared to non-users among clients of the public dental care clinics in Stockholm region during the second epidemic wave (October 1, 2020 – June 30, 2021).

		Diagnoses of Covid-19	Hospital admission	Intensive care	Death
		Adjusted[a] RR (95% CI)	Adjusted[a] RR (95% CI)	Adjusted[a] RR (95% CI)	Adjusted[a] RR (95% CI)
**Females**	**Current tobacco Use**				
	Non-user of snus	(ref)	(ref)	(ref)	(ref)
	Snus user (all)	1.08 (1.03-1.14)	1.17 (0.81-1.69)	1.27 (0.38-4.28)	NE
	<1/2 can/day	1.09 (1.03-1.16)	1.27 (0.82-1.95)	1.84 (0.55-6.18)	NE
	>½ cans/day	1.05 (0.96-1.15)	0.95 (0.48-1.91)	NE	NE
**Males**	**Current tobacco Use**				
	Non-user of snus	(ref)	(ref)	(ref)	(ref)
	Snus user (all)	1.14 (1.10-1.18)	1.10 (0.93-1.30)	1.23 (0.77-1.98)	0.71 (0.32-1.57)
	<1/2 can/day	1.12 (1.08-1.17)	1.07 (.87-1.32)	1.06 (0.54-2.07)	0.40 (0.10-1.56)
	>½ cans/day	1.16 (1.11-1.21)	1.14 (0.90-1.43)	1.47 (0.80-2.68)	1.26 (0.51-3.13)

[a] Adjusted for smoking, sex, age, education, income, occupational risk, country of birth and cohabitation. *NE* not estimated because of the low number of events.

Supplementary Table S3. Adjusted Relative Risk (RR) and 95% Confidence Intervals (CI) of COVID-19 for current dual users of tobacco (smokers and snus users), exclusive smokers, and exclusive snus users compared to non-users of tobacco among clients of the public dental care clinics in Stockholm region during the second epidemic wave (October 1, 2020 – June 30, 2021).

		Diagnoses of Covid-19	Hospital admission	Intensive care	Death
		Adjusted[a] RR (95% CI)	Adjusted[a] RR (95% CI)	Adjusted[a] RR (95% CI)	Adjusted[a] RR (95% CI)
**Current tobacco Use**					
Non user		(ref)	(ref)	(ref)	(ref)
Exclusive smokers		0.78 (0.75-0.81)	0.67 (0.56-0.81)	0.71 (0.41-1.23)	0.78(0.40-1.52)
Exclusive *Snus* Users		1.12 (1.09-1.15)	1.10 (0.95-1.28)	1.19 (0.76 -1.85)	0.66 (0.30-1.44)
Smokers and Snus users		0.80 (0.74–0.87)	0.68 (0.41-1.12)	0.70 (0.17-2.85)	0.91 (0.12-6.56)

[a] Adjusted for sex, age, education, income, occupational risk, country of birth and cohabitation.

Supplementary Table S4. Adjusted Relative Risk (RR) and 95% Confidence Intervals (CI) of COVID -19 diagnoses for exclusive smokers compared to non-tobacco users among clients of the public dental care clinics in Stockholm region during the second epidemic wave (October 1, 2020 – June 30, 2021), stratified by period of assessment of tobacco use.

	**Tobacco use assessed 2019-20 N= 273,505**				**Tobacco use assessed 2018 N= 80,694**				**Tobacco use assessed 2015-17 N= 64,660**			
	Diagnoses of Covid-19	Hospital admission	Intensive care	Death	Diagnoses of Covid-19	Hospital admission	Intensive care	Death	Diagnoses of Covid-19	Hospital admission	Intensive care	Death
**Current tobacco Use**												
Non user												
Smoker (all)	(ref)	(ref)	(ref)	(ref)	(ref)	(ref)	(ref)	(ref)	(ref)	(ref)	(ref)	(ref)
<=10 cig/day	0.77 (0.73-0.81)	0.68 (0.54-0.86)	0.66 (0.33-1.31)	0.64 (0.26-1.57)	0.82 (0.76–0.89)	0.69 (0.45-1.08)	NE	NE	0.77 (0.71–0.84)	0.57 (0.37-0.87)	NE	NE
>10 cig/day	0.84 (0.80-0.89)	0.78 (0.59-1.02)	0.49 (0.18-1.34)	0.86 (0.32-2.33)	0.90 (0.82–0.99)	0.79 (0.47-1.33)	NE	NE	0.88 (0.80–0.96)	0.70 (0.42-1.16)	NE	NE
	0.62 (0.56-0.67)	0.53 (0.35-0.79)	0.90 (0.36-2.24)	0.32 (0.04-2.29)	0.65 (0.55-0.76)	0.55 (0.26-1.17)	NE	NE	0.58 (0.50-0.68)	0.41 (0.20-0.84)	NE	NE

[a] Adjusted for sex, age, education, income, occupational risk, country of birth and cohabitation. *NE* not estimated because of the low number of events.

Supplementary Table S5. Adjusted Risk Ratio (RR) and 95% Confidence Intervals (CI) of COVID-19 for current exclusive *snus* users compared to non-tobacco users among clients of the public dental care clinics in Stockholm region, stratified by period of assessment of tobacco use.

		**Tobacco use assessed 2019-20 N= 273,505**				**Tobacco use assessed 2018 N= 80,694**				**Tobacco use assessed 2015-17 N= 64,660**			
		Diagnoses of Covid-19	Hospital admission	Intensive care	Death	Diagnoses of Covid-19	Hospital admission	ICU	Death	Diagnoses of Covid-19	Hospital admission	Intensive care	Death
**Females**	**Current tobacco Use**												
	Non user												
	Snus user (all)	(ref)	(ref)	(ref)	(ref)	(ref)	(ref)	(ref)	(ref)	(ref)	(ref)	(ref)	(ref)
	<1/2 can/day	1.14 (1.08-1.22)	1.37 (0.90-2.09)	1.87 (0.53-6.57)	NE	0.98 (0.87-1.11)	0.92 (0.38-2.28)	NE	NE	0.92 (0.80-1.06)	0,51 (0.12-2.11)	NE	NE
	>½ cans/day	1.16 (1.08-1.25)	1.55 (0.96-2.48)	NE	NE	1.01 (0.88-1.16)	0.81 (0.26-2.54)	NE	NE	0.89 (0.75-1.05)	0.76 (0.18-3.09)	NE	NE
		1.10 (0.99-1.23)	0.97 (0.40-2.34)	NE	NE	0.92 (0.75-1.14)	1.18 (0.29-4.83)	NE	NE	0.99 (0.79-1.24)	NA	NE	NE
**Males**	**Current tobacco Use**												
	Non user												
	Snus user (all)	(ref)	(ref)	(ref)	(ref)	(ref)	(ref)	(ref)	(ref)	(ref)	(ref)	(ref)	(ref)
	<1/2 can/day	1.17 (1.12-1.21)	1.24 (1.02-1.50)	1.25 (0.72-2.19)	0.39 (0.12-1.26)	1.07 (0.99-1.14)	1.05 (0.68-1.61)	NE	NE	1.13 (1.05-1.21)	0.63 (0.38-1.03)	NE	NE
	>½ cans/day	1.16 (1.11-1.22)	1.10 (0.86-1.42)	0.95 (0.44-2.08)	0.20 (0.03-1.46)	1.04 (0.95-1.14)	1.37 (0.84-2.23)	NE	NE	1.13 (1.02-1.24)	0.57 (0.29-1.14)	NE	NE
		1.17 (1.10-1.23)	1.42 (1.09-1.83)	1.67 (0.81-3.43)	0.73 (0.17-3.11)	1.10 (1.00-1.21)	0.65 (0.30-1.40)	NE	NE	1.12 (1.02-1.24)	0.69 (0.36-1.33)	NE	NE

[a] Adjusted for sex, age, education, income, occupational risk, country of birth and cohabitation. *NE* not estimated because of the low number of events.

Supplementary Table S6. Adjusted Risk Ratio (RR) and 95% Confidence Intervals (CI) of mutually exclusive outcome events (any diagnosis of COVID-19 that did not result in hospital admission, ICU, or death; diagnoses that resulted in hospital admission but not ICU or death; diagnoses resulting in ICU admission, but not death; death) for current users compared to non-users of tobacco among clients of the public dental care clinics in Stockholm region during the second epidemic wave (October 1, 2020 – June 30, 2021).

		**Diagnoses that did not result in hospital admission, ICU, or death**	**Diagnoses that resulted in hospital admission, but not ICU or death**	**Diagnoses resulting in ICU admission, but not death**	**Death**
		Adjusted[a] RR (95% CI)	Adjusted[a] RR (95% CI)	Adjusted[a] RR (95% CI)	Adjusted[a] RR (95% CI)
**Current tobacco Use**					
Non user		(ref)	(ref)	(ref)	(ref)
Exclusive smokers		0.79 (0.76-0.82)	0.99 (0.82-1.19)	1.04 (0.57-1.91)	0.91 (0.46-1.83)
Exclusive *snus* Users		1.13 (1.10-1.16)	1.06 (0.91-1.25)	1.34 (0.84-2.13)	0.73 (0.34-1.54)
Smokers and *Snus* Combined		0.81 (0.75-0.89)	0.98 (0.58-1.63)	0.59 (0.08-4.22)	1.45 (0.20-10.54)

[a] Adjusted for sex, age, education, income, occupational risk, country of birth and cohabitation

Supplementary Table S7. Adjusted Risk Ratio (RR) and 95% Confidence Intervals (CI) of COVID-19 for current exclusive smokers compared to non-tobacco users among clients of the public dental care clinics in Stockholm region in pre-and post-vaccination periods during the second epidemic wave (October 1, 2020 – June 30, 2021).

		**Diagnoses of COVID-19**	**Hospital admission**	**Intensive care**	**Death**
		Adjusted[a] RR (95% CI)	Adjusted[a] RR (95% CI)	Adjusted[a] RR (95% CI)	Adjusted[a] RR (95% CI)
**Pre-vaccination (October 1, 2020 – December 31, 2020)**					
**Current tobacco Use**					
Non user		(ref)	(ref)	(ref)	(ref)
Smoker (all)		0.74 (0.70–0.78)	0.66 (0.49-0.87)	NE	1.00 (0.40-2.46)
<=10 cig/day		0.86 (0.80–0.91)	0.86 (0.62-1.18)	NE	1.00 (0.32-3.11)
>10 cig/day		0.49 (0.44-0.55)	0.37 (0.21-0.65)	NE	1.01 (0.24-4.18)
**Post-vaccination (January 1, 2021 – June 30, 2021)**					
**Current tobacco Use**					
Non user		(ref)	(ref)	(ref)	(ref)
Smoker (all)		0.79 (0.76–0.83)	0.64 (0.51-0.82)	0.79 (0.41-1.52)	0.73 (0.32-1.70)
<=10 cig/day		0.85 (0.80–0.90)	0.69 (0.51-0.92)	0.51 (0.18-1.40)	0.64 (0.20-2.06)
>10 cig/day		0.69 (0.63–0.75)	0.58 (0.40-0.85)	1.17 (0.52-2.61)	0.86 (0.27-2.75)

[a] Adjusted for sex, age, education, income, occupational risk, country of birth and cohabitation. NE not estimated because of the low number of events.

Supplementary Table S8. Adjusted Risk Ratio (RR) and 95% Confidence Intervals (CI) of diagnoses of COVID-19 for current exclusive *snus* users compared to non-users of tobacco among clients of the public dental care clinics in Stockholm region in pre-and post-vaccination periods during the second epidemic wave (October 1, 2020 – June 30, 2021).

		Diagnoses of COVID-19	Hospital admission	Intensive care	Death
		Adjusted[a] RR (95% CI)	Adjusted[a] RR (95% CI)	Adjusted[a] RR (95% CI)	Adjusted[a] RR (95% CI)
**Pre-vaccination (October 1, 2020 – December 31, 2020)**					
**Females**	**Current tobacco Use**				
	Non-Tobacco User	(ref)	(ref)	(ref)	(ref)
	Snus user (all)	1.12 (1.04-1.21)	1.02 (0.55-1.86)	NE	NE
	<1/2 can/day	1.13 (1.03-1.24)	1.07 (0.53-2.16)	NE	NE
	>½ cans/day	1.11 (0.97-1.27)	0.90 (0.28-2.82)	NE	NE
**Males**	**Current tobacco Use**				
	Non-Tobacco User	(ref)	(ref)	(ref)	(ref)
	Snus user (all)	1.16 (1.11-1.22)	1.24 (0.96-1.60)	NE	NE
	<1/2 can/day	1.15 (1.08-1.23)	1.10 (0.79-1.54)	NE	NE
	>½ cans/day	1.18 (1.10-1.26)	1.42 (1.01-2.01)	NE	NE
**Post-vaccination (January 1, 2021 – June 30, 2021)**					
**Females**	**Current tobacco Use**				
	Non-Tobacco User	(ref)	(ref)	(ref)	(ref)
	Snus user (all)	1.04 (0.97-1.12)	1.26 (0.80-1.98)	NE	NE
	<1/2 can/day	1.06 (0.98-1.16)	1.46 (0.88-2.42)	NE	NE
	>½ cans/day	1.00 (0.88-1.13)	0.80 (0.30.2.15)	NE	NE
**Males**	**Current tobacco Use**				
	Non-Tobacco User	(ref)	(ref)	(ref)	(ref)
	Snus user (all)	1.14 (1.09-1.19)	1.01 (0.82-1.25)	0.79 (0.40-1.56)	0.91 (0.38-2.17)
	<1/2 can/day	1.14 (1.08-1.20)	1.03 (0.79-1.35)	0.98 (0.45-2.14)	0.73 (0.22-2.38)
	>½ cans/day	1.14 (1.08.1.21)	0.98 (0.72.1.33)	0.53 (0.16-1-74)	1.21 (0.36-4.02)

[a] Adjusted for age, education, income, occupational risk, country of birth and cohabitation. NE not estimated because of the low number of events.
